# Dual-source CT coronary angiographic evaluation of coronary artery fistulas

**DOI:** 10.3892/etm.2014.1602

**Published:** 2014-03-04

**Authors:** MIN LIU, QING HOU, XIAOJUAN GUO, SHUANGKUN WANG, ZHANHONG MA

**Affiliations:** 1 Department of Radiology, Beijing Chaoyang Hospital, Capital Medical University, Beijing 100020, P.R. China; 2 Department of Radiology, Beijing Puren Hospital, Beijing 100069, P.R. China

**Keywords:** coronary artery fistula, dual-source computed tomography, computed tomography coronary angiography

## Abstract

The aim of the present study was to retrospectively evaluate the incidence and morphological features of coronary artery fistulas (CAFs) detected by dual-source computed tomography coronary angiography (DS-CTCA). Between January 2011 and January 2013, 19,584 consecutive patients that had undergone electrocardiogram-triggering DS-CTCA were retrospectively reviewed. Image reconstructions were performed and image quality was evaluated. The medical information of the patients with CAF was reviewed from the medical records. Among the 19,584 patients, 66 patients were diagnosed with CAFs by CTCA, including 60 patients with coronary pulmonary artery fistulas (CPAFs) and six with coronary left ventricular fistulas. Therefore, the incidence of CAFs was 0.34%. Image quality was considered to be excellent in 61 patients and moderate in five cases. CPAFs were identified as small and tortuous vessels in 24 patients and dilated vessels close to the surface of the pulmonary artery (PA) in 36 patients. The coronary left ventricular fistulas were identified as dilated vessels that were draining into the posterior wall of the left ventricle. Among the 66 patients, 54 patients had one traceable fistula and the remaining 12 patients were shown to have two fistula vessels. The average diameter of the detected fistulas, measured with CTCA, was 3.1±1.9 mm. A high-density flow jet of contrast agent shunting from the fistula into the low density PA was observed in 46 patients with CPAF. The results indicate that DS-CTCA is a reliable noninvasive tool that allows the accurate delineation of CAFs.

## Introduction

Coronary artery fistulas (CAFs), defined as abnormal vascular communications between any coronary artery and any of the cardiac chambers or great vessels, are generally noticed incidentally on diagnostic cardiac catheterization in the adult population (1). CAF is a type of rare congenital coronary anomaly. Its actual incidence is unknown. Symptoms are mainly dependent on the severity of left-to-right shunt. The majority of the adult cases are generally asymptomatic and rare, but certain cases cause severe life-threatening events. At present, various imaging modalities are available for coronary artery assessment. Due to the complex structural anatomy and the probability of the multiple fistulas arising from different segments of the coronary arteries and coronary sinuses, conventional coronary angiography (CAG) may not be sufficient. Temporal and spatial resolution of MR angiography and echocardiography was inferior to that of CAG. Computer tomography coronary angiography (CTCA) not only non-invasively demonstrates the origin, structural anatomy of coronary, but also is easy to follow-up (2). CTCA has been the most important method for evaluating the coronary artery diseases. Dual-source computed tomography (DSCT), with two arrays consisting of an X-ray tube and detectors arranged at a 90° angle and a gantry rotation time of 330 msec, allows temporal resolution of 83 msec and provides higher image quality compared with multi-detector CT. At present, few reports focus on the value of DSCT evaluation of CAFs. In the present study, we aim to evaluate the incidence and morphologic features, imaging quality of CAF by the dual-source CT coronary angiography (DSCT).

## Materials and methods

### Patients

In total, 19,584 consecutive patients that had undergone CTCA between January 2011 and January 2013 in two imaging centers (The Department of Radiology of Beijing Chaoyang Hospital and The Department of Radiology of Beijing Puren Hospital, Beijing, China) were retrospectively screened. Each CTCA image was reviewed by two radiologists using a Picture Archiving Communication System (Radiology RA 1000 Worksation; GE Healthcare, Fairfield, CT, USA) and 66 patients were diagnosed with CAFs. Next, the medical records of these patients were reviewed retrospectively. The ethics committee of Beijing Chaoyang Hospital and Beijing Puren Hospital approved the study and written informed consent was provided by all patients.

### DS-CTCA

The DS-CT system (SOMATOM Definition; Siemens Healthcare, Erlangen, Germany) was used to scan all patients. All CTCA procedures were performed without heart rate (HR) modulation by administrating β-blockers. A mechanical injector was used for intravenous bolus injections of 370 mg/ml iopromide (Ultravist; Bayer Healthcare Pharmaceuticals, Berlin, Germany) at a flow rate of 5.0 ml/sec. Coronary contrast was controlled by bolus tracking in the ascending aorta (signal attenuation threshold, 120 HU). All injections were followed by further injections of 50 ml saline. CTCA was performed with the prospective electrocardiogram (ECG)-triggering or the retrospective ECG-triggering protocols, according to HR of the patient. If the HR was <70 bpm, the prospective ECG-triggering scan mode was selected, but if the HR was ≥70 bpm, the retrospective ECG-triggering scan mode was used. The ECG-triggering CTCA scanned with the following parameters: Detector collimation, 2×32×0.6 mm; slice acquisition, 2×64×0.6 mm by the means of a z-flying focal spot; and gantry rotation time, 330 msec. Tube current was adapted automatically to the weight of each patient using CARE Dose 4D automatic exposure control (Siemens Healthcare) and a reference tube current of 320 mAsec was used. A tube voltage of 120 kV was selected when the body mass index (BMI) of the patient was ≥24 kg/m^2^, while 100 kV was selected when the BMI was <24 kg/m^2^. For the retrospective ECG-triggering protocol, the ECG-pulsing window was set at 35–75% of the RR interval with a pitch of 0.2–0.43, which was automatically adapted to the HR.

### Image quality and reconstruction

All CTCA images were transferred to a dedicated workstation (ADW 4.2; GE Healthcare) and reconstructed by a cardiovascular radiologist with ten years’ experience. CAF was assessed by two radiologists with consensus. Following the evaluation of axial and oblique multiplanar reconstructions, other rendering methods were used to create images, including three-dimensional volume-rendered (VR), curved multiplanar and maximum intensity projection (MIP) images. Image quality was assessed using the following four point scale: Excellent, no artifacts, unrestricted evaluations of the fistula; good, minor artifacts, good diagnostic quality; adequate, moderate artifacts, acceptable diagnostic quality; unacceptable, severe artifacts impairing accurate evaluation. The origin vessels, draining veins, presence of aneurysm, combined congenital or acquired anomaly and the relationship with adjacent structures were assessed from the axial and the reformatted images together.

## Results

### Diagnosis of CAFs

Among the 19,548 cases, CAFs were diagnosed in 66 patients (female, 37; male, 29; age, 35–79 years, mean, 58.1±10.1 years), an incidence of 0.34% (66/19,548). A total of 52 patients were examined by the retrospective ECG-triggering protocol. For this method the mean dose length product (DLP) was 611.0±266.5 mGy·cm, corresponding to an effective dose estimation of 13.8±5.1 mSv. The remaining 14 patients were examined by the prospective ECG-triggering protocol in the 70%-RR interval (mean HR, 58±5 bpm). The mean DLP was 207.3±57.7 mGy·cm, which corresponded to an effective dose estimation of 4.1±1.9 mSv. The image quality was excellent for 61 patients and moderate for 5 patients. None of the images were considered to be of adequate or unacceptable quality.

### Diagnosis of coronary pulmonary artery fistulas (CPAFs) and coronary left ventricular fistulas

Among the 66 patients with CAFs, 60 patients were diagnosed with having a CPAF (female, 35; male, 25; age, 39–79 years, mean, 58.1±10.5 years) and the remaining six patients were diagnosed with a coronary left ventricular fistula (female, 2; male, 4; age, 54–65 years). The incidence of CPAF was 0.31% (60/19,548). Invasive CAG was performed in 10 patients (female, 3; male, 7; age, 51–72 years), which included six cases of CPAFs and four cases of coronary left ventricular fistulas. The CTCA and CAG observations of these 10 patients are summarized in Table I. Four patients with CPAFs were treated with fistula coiling.

### Clinical presentations

Among the 66 patients, 40 patients undergoing CTCA presented with chest pain, five patients presented with chest tightness and syncope and one patient had heart failure. Six cases had already been diagnosed with coronary artery disease, four cases were under follow-up observation and 10 patients were suspected of ischemic heart disease from other tests. Using CTCA, 36 patients were diagnosed with isolated CAFs without other cardiac diseases or malformations, 25 patients were found to have lipid or calcified plaques, four patients had coronary myocardial bridges and two patients had undergone coronary angioplasty. DS-CTCA observations are summarized in Table II.

### Fistula locations

**Figure 1:**
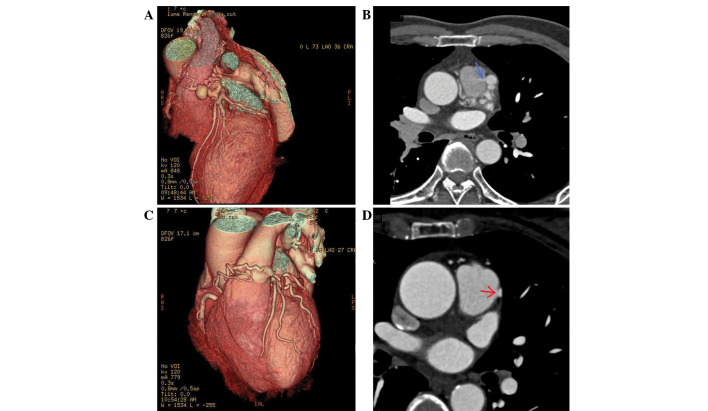
(A) VR image shows an aneurysm and tortuous vessel communicating with the proximal LCA and the PA in a 51-year-old male. (B) MIP image shows a high-density jet flow (blue arrow) directly injecting into the main PA. (C) VR image shows an aneurysm and tortuous vessel communicating with the proximal RCA and the PA in a 62-year-old female. (D) Axial image shows a PA artery wall defect (red arrow). LCA, left coronary artery; RCA, right coronary artery; VR, volume rendered; PA, pulmonary artery; MIP, maximum intensity projection.

In 24 patients, CPAFs were identified as small and tortuous vessels (Fig. 1), while in 36 patients, CPAFs were identified as dilated vessels close to the surface of the pulmonary artery (PA). The drainage sites were located on the left lateral side of the pulmonary trunk in 54 patients and on the anterior side of the pulmonary trunk in six patients. Small aneurysms of fistula vessels were identified in 11 patients (Fig. 1A). The mean diameter of the detected fistulas, measured with CTCA, was 3.1±1.9 mm (range, 1.4–13.3 mm). A high-density flow jet of contrast agent shunting from the fistula into the low density PA was observed in 46 CPAF cases(Fig. 1B) and small defects of the PA wall, without shunting flow jet, were observed in 14 patients (Fig. 1C and D).

**Figure 2:**
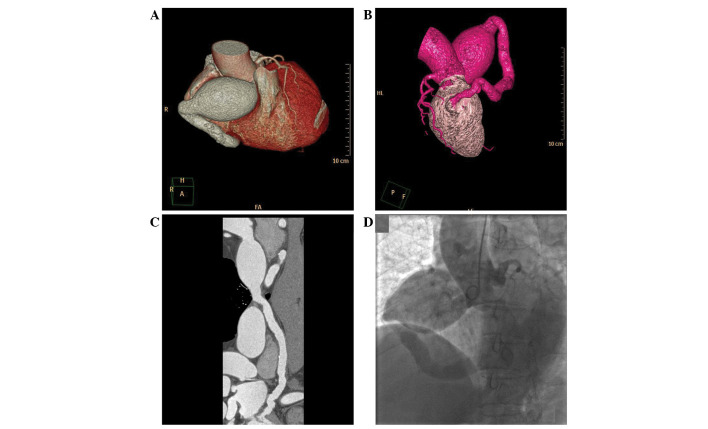
CAF originating from the RCA and draining into the LV in a 65-year-old female with heart failure. VR images show (A) a huge aneurysm in the proximal segment of the RCA and (B) a fistula vessel draining into the LV. (C) Curved multi-planar image shows a shunt from the RCA to the posterior wall of the LV. (D) CAG image shows communication between the RCA and the LV. CAF, coronary artery fistula; LV, left ventricle; RCA, right coronary artery; VR, volume rendered; CAG, coronary angiography.

From the CTCA images, coronary left ventricular fistulas in six patients were identified in the dilated vessels draining into the posterior wall of the left ventricle (LV). Moreover, a large right coronary aneurysm was observed in one patient with a large fistula of the right coronary artery (RCA) to the LV (Fig. 2).

### Fistula origins

In the 66 patients with CAFs, 54 patients had one fistula that could be traced and the remaining 12 patients were shown to have two fistula vessels. It was shown that 31 cases (47.0%) originated from the left coronary artery (LCA), 26 cases (39.4%) originated from the RCA and 9 cases (13.6%) originated from the LCA and RCA. Among the 60 CPAF patients, 29 cases (48.3%) originated from the LCA, 22 cases (36.7%) originated from the RCA and nine cases (15%) originated from the LCA and RCA. Among the 29 cases that originated from the LCA, 27 cases were found to originate from the left anterior descending artery (LAD) and two cases from the left main artery (LMA). Among the nine cases originating from the LCA and RCA, two cases originated from the LMA and proximal RCA and seven cases originated from the LAD and proximal RCA. Among the six detected coronary left ventricular fistula cases, the fistula vessels were found to originate from the RCA in four patients and the LAD in two patients and lead to the LV.

## Discussion

In total, 19,548 patients that had undergone DS-CTCA were included in the study, which to the best of our knowledge is the largest study cohort from two centers. Only 66 patients with CAFs were detected using CTCA, which was an incidence rate of 0.34%. CAFs are rare congenital malformations that are highly variable. The majority of cases are generally asymptomatic; however, specific cases can cause severe life threatening events (4,5). CAFs were accidentally identified during routine cardiac catheterization in patients suspected of having atherosclerotic coronary artery disease and since then CAG has been used as the standard modality for diagnosis (6). The incidence of CAFs in the adult population is reported to be 0.05% (7). In the present study, 66 patients (66/19,548) were diagnosed with CAFs using CTCA and only 10 of the patients (10/66) with CAFs were examined by CAG for the investigation of significant symptoms and the other 56 patients choose a long-time follow-up. Therefore, the true incidence of CAFs is highly speculative since numerous CAF cases are symptomless and may not be detected. The present study revealed the incidence of CPAFs to be 0.31% using DS-CTCA, which is consistent with the incidence of 0.32% indicated by 64-slice multidetector-CT (2).

DS-CT performed with two arrays consisting of an X-ray tube, detectors arranged at a 90° angle and a gantry rotation time of 330 msec, allows temporal resolution of 83 msec and provides higher image quality compared with that of multidetector-CT (3). The scanning mode was selected according to the HR and BMI of the patients. Patients with significant arrhythmia were not recommended to undergo a CTCA scan. In the present study, the image quality was not considered to be unacceptable for any of the patients, despite HR not being controlled prior to CTCA. A total of 52 patients were examined by the retrospective ECG-triggering protocol and 14 patients were examined by the prospective ECG-triggering protocol. The prospective method uses a significantly lower radiation dose compared with that used by the retrospective ECG-gated technique. This indicates that the prospective ECG-triggering protocol provides the clear anatomy of CAFs with a lower radiation dose. Moreover, the use of multiplanar reformatted images clearly demonstrates the site of origin, the termination of abnormal blood vessels and small defects of the pulmonary wall. VR images provided an excellent overview of the cardiac and vascular anatomy. From the MIP images, a high-density flow jet of contrast agent shunting from the fistula into the low density PA was clearly observed and small, tortuous or dilated vessels close to the surface of the PA were clearly demonstrated in VR images. These were the direct manifestations of CAFs in CTCA.

There have been numerous types of CAF reported. Over 90% of fistulas reported drain into the venous circulation and the most common drainage site is the right ventricle (8). CPAFs constitute 15–30% of all CAF cases (9). However, Kim *et al* (2) indicated that CPAFs account for 89.5% of CAF cases. In the present study, the most common CAF was CPAF, which is consistent with the study by Yun *et al* (10). The other six patients were diagnosed with coronary left ventricular fistulas. The present study indicated that CPAFs accounted for 91% of the CAF cases.

It has been reported (11) that ~50% of CAF cases arise from the RCA, ~42% from the LCA and ~5% from the RCA and LCA. In the present study, the CAFs originated from the RCA in 39.4% of patients, the LCA in 47.0% and the RCA and LCA in 13.6% of patients. The incidence of CAFS arising from the RCA and LCA in the present study is less than the 22.9% reported by Yun *et al* (10). In the CPAF group, the most common drainage coronary artery was the LAD followed by the proximal RCA and then the LMA.

Clear guidelines for the treatment of CPAFs have not yet been established. According to Liberthson *et al* (12), patients ≥20 years old who have undergone coronary arteriovenous fistula ligation have an increased probability of complications (23%), including postoperative mortality (7%) and myocardial infarction (7%). However, in the present study, there were no patients <20 years-old and the patients that received treatment did not develop any of the aforementioned problems.

The main limitation of the present study is that it is a retrospective study. Although the image quality was acceptable for each case, the majority of cases underwent CTCA with the retrospective ECG-triggering protocol, leading to a higher radiation dose compared with that used in the prospective ECG-triggering method. A number of studies have indicated that the prospective ECG-triggering protocol is successful in providing acceptable image quality with a significant reduction of radiation dose, even in patients with a high HR. Only 14 patients in the current study were scanned by this method and the CAFs were depicted clearly with a lower radiation dose. Therefore, the prospective ECG-triggering protocol should be used for evaluating CAFs in future practice. Patients who underwent CTCA were suspected of having coronary artery disease. However, the majority of cases of CAF are generally asymptomatic. This indicates that the true incidence of CAF in the population is not clear.

In conclusion, the incidence of CAFs detected by DS-CTCA in the present study was 0.34%. DS-CTCA is a reliable noninvasive tool that allows accurate the delineation of CAFs and provides detailed three-dimensional anatomical information.

## Figures and Tables

**Table I:** CTCA and CAG observations of patients with CAFs.

Patient	Age, years	Gender	CTCA observations	CAG observations
1	63	F	LAD-PA	LAD-PA Transcatheter closure of coronary artery to pulmonary artery coil
2	56	M	LAD-PA-RCA	LAD-PA-RCA
3	61	M	LMA-PA	LMA-PA Transcatheter closure of coronary artery to pulmonary artery coil
4	67	M	RCA-PA	RCA-PA Transcatheter closure of coronary artery to pulmonary artery coil
5	64	M	LAD-PA-RCA	LAD-PA-RCA
6	51	M	LMA-PA	LMA-PA Transcatheter closure of coronary artery to pulmonary artery coil
7	54	M	LAD-LV	LAD-LV
8	68	F	RCA-LV	RCA-LV
9	72	M	LAD-LV	LAD-LV
10	65	F	RCA-LV	RCA-LV

CTCA, computed tomography coronary angiography; CAG, coronary artery angiography; CAF, coronary artery fistula; LAD, left anterior descending artery; PA, pulmonary artery; LMA, left main artery; RCA, right coronary artery; LV, left ventricle; F, female; M, male.

**Table II:** CTCA observations of patients with CAFs.

CTCA manifestation	Patients, n
CAF	66
CPAF	60
Coronary left ventricular fistula	6
Isolated CAF	36
Isolated CPAF	35
Isolated coronary left ventricular fistula	2
Lipid or calcified plaque	25
Coronary artery stenosis >50%	5
Percutaneous transluminal coronary angioplasty	2
Myocardial bridging	4
Coronary aneurysm	1
Permanent left superior vena cava	1

CTCA, computed tomography coronary angiography; CAF, coronary artery fistula; CPAF, coronary pulmonary artery fistula; CAG, coronary angiography.
